# Prolactinoma presenting as chronic anaemia with osteoporosis: a case report

**DOI:** 10.1186/1752-1947-4-33

**Published:** 2010-02-01

**Authors:** Fergus R MacLean, John P Hanley

**Affiliations:** 1 Department of Bone Health, Diabetes and Endocrinology, Southern General Hospital, Govan Road, Glasgow, G51 4TF, UK; 2 Department of Haematology, Royal Victoria Infirmary, Queen Victoria Road, Newcastle Upon Tyne, NE1 4LP, UK

## Abstract

**Introduction:**

Unexplained anaemia is a rare mode of presentation for prolactinoma. We describe a case of a man, with chronic anaemia ascribed to old age. Six years later, he was evaluated and diagnosed with a prolactinoma and resultant osteoporosis. Prolactinoma in old people may present insidiously with chronic anaemia and osteoporosis with or without sexual dysfunction.

**Case presentation:**

We describe the case of a 70-year-old Caucasian man who presented with mild anaemia and tiredness. His anaemia was investigated and ascribed to senescence. Endocrine causes were not considered or tested for. Six years later, he was again referred. Reassessment and direct questioning revealed long-standing sexual dysfunction. It was also discovered that our patient had fractured his radius twice, with minor trauma, during the preceding year. His serum prolactin was massively increased and a magnetic resonance imaging (MRI) scan of the head demonstrated a pituitary mass consistent with a prolactinoma. Dual X-ray absorptiometry revealed osteoporosis. Treatment of the prolactinoma led to a reduction in his serum prolactin with a rise in his haemoglobin to normal levels. This suggested that the prolactinoma was present during the initial presentation and was the cause of his anaemia.

**Conclusion:**

This case highlights the importance of fully evaluating and investigating unexplained anaemia in older people and that endocrine causes should be considered. Osteoporosis also requires evaluation with secondary causes considered.

## Introduction

Unexplained anaemia is a rare mode of presentation for prolactinoma. We describe a 70-year-old man whose diagnosis of prolactinoma was not revealed until after six years. During the initial evaluation, his anaemia was ascribed to old age. However, after six years, prolactinoma was diagnosed and subsequently successfully treated with cabergoline, thus reversing his anaemia and its symptoms.

## Case presentation

A 70-year-old Caucasian man with mild chronic anaemia and tiredness was referred to the haematology. Six years before, he had been referred to secondary care. At the time of the initial assessment, he was noted to have a normochromic normocytic anaemia with a haemoglobin (Hb) of 109 g/L, mean corpuscular volume 84fL, platelet count 307 × 10^9^/L, and white cell count (WCC) of 7.9 × 10^9^/L with a normal differential. Serum vitamin B12, red cell folate and iron studies were normal. Despite this, he was investigated with an upper gastrointestinal endoscopy, sigmoidoscopy, barium enema and abdominal ultrasound - all of which were normal. Endocrine causes for the anaemia were not considered. The anaemia was ascribed to old age. He was not followed up but he presented to primary care six years later with ongoing tiredness. He was found to have a mild anaemia (Hb 118 g/L, WCC 5.6 × 10^9^/L, platelets 330 × 10^9^/L). Because of his anaemia and tiredness, it was decided that he be referred to haematology. Haematology review showed that the blood film of our patient was normochromic and normocytic and that serum vitamin B12, red cell folate, iron studies, liver function tests, routine biochemistry and serum protein electrophoresis were all normal. During clinic review, direct questions revealed that our patient had already noticed the gradual onset of sparse body hair with loss of libido and erections six years before, during the time of his first presentation. He had ascribed these changes to his age and considered them unimportant. Further questioning revealed that in the year prior to the haematology review he had fractured his left radius on two separate occasions with minimal trauma. On examination, he appeared pale with sparse axillary and pubic hair with no galactorrhoea or gynaecomastia. On neurological examination there were no visual field defects or cranial nerve abnormalities.

The serum prolactin was 31000 mIU/L (20-300), luteinising hormone (LH) 0.3 IU/L (2-9), follicle stimulating hormone (FSH) 0.6IU/L (2-9) and testosterone 0.8 nmol/L (8 -38). The random cortisol was 237 nmol/L with a cortisol of 540 nmol/L 1hour post intramuscular injection with 250 μg of tetracosactrin. Insulin-like growth factor was 60 μg/L (58-227). The thyroid stimulating hormone was 1.65 mU/L (0.25-2.5) and free thyroxine index 37 (55-160). Pituitary MRI demonstrated a pituitary mass (20 mm vertically) consistent with a macroadenoma. Formal perimetry revealed no visual field defect. Bone mineral density (evaluated by dual X-ray absorptiometry) confirmed osteoporosis (t score L2-L4 -2.9 standard deviations, t score right neck of femur -3.1 standard deviations). Our patient was commenced on cabergoline rising to a dose of 2 mg weekly. After one year, he continued with 2 mg of cabergoline weekly. Six months after starting cabergoline, his Hb had risen to 126 g/L. A year after commencing cabergoline, his prolactin fell to 923 mIU/L and his Hb rose to 137 g/L with a dissipation of his symptoms.

## Discussion

The clinical presentation of prolactinoma depends on patient's age, gender and duration of hyperprolactinaemia [1]. Women of reproductive age tend to present with menstrual disturbance, infertility, delayed menarche or galactorrhoea. Loss of libido, impotence (partial or complete) and infertility are possible presenting features in younger males [1]. Hyperprolactinaemia can create a hypogonadic state [2] which is associated with anaemia. However, an unexplained anaemia is a rare mode of presentation for prolactinoma. In the case described above, the mild anaemia was extensively investigated during his first presentation. But endocrine causes were not considered or tested for, and anaemia was merely ascribed to old age. Six years later, the anaemia was resolved when the prolactin levels were reduced, making it reasonable to deduce that hyperprolactinaemia was the cause of the anaemia all along.

It is believed that hyperprolactinaemia impairs pulsatile gonadotrophin (LH and FSH) release by interfering with hypothalamic gonadotrophin releasing hormone secretion [1]. The resulting hypogonadism, with reduced serum testosterone, is reversible with the reduction of prolactin secretion [2]. Hypogonadism may also be caused by a direct pressure effect from an enlarging prolactinoma in the context of a hypopituitary state. Androgens stimulate erythropoeisis, at least in part, by increasing levels of erythropoetin [3]. During puberty, the male haemoglobin concentration rises above that of the female which is not related to iron deficiency, pregnancy or blood loss [3]. Osteoporosis is also a well-recognised complication of prolonged hypogonadism in men [4,5]. Slightly lower haemoglobin values may be encountered by very old people [6], who have been thought to present a physiological response of senescence [7]. However, they are not statistically significant. Healthy older men and women should have haematological values similar to younger adults [6]. Our patient was at the age when complaints of sexual dysfunction are rare, unless symptoms are directly sought in them. Presentation of prolactinoma in the elderly persons may simply be as anaemia and tiredness with or without osteoporosis.

## Conclusion

Anaemia in the elderly people should be fully evaluated, with endocrine causes considered. Symptoms of hypogonadism may not be found unless directly sought. Osteoporosis may be secondary in origin and this should be kept in mind when assessing patients of all ages. In the older person, prolactinoma may present insidiously as a chronic anaemia and hypogonadism with or without osteoporosis.

## Consent

At the time of the diagnosis, written informed consent was gained from our patient for publication of this case report. Every effort was made to gain up to date consent from our patient for this publication but this has not been possible. The identity of our patient will remain anonymous, making publication possible without contemporaneous consent.

## Competing interests

The authors declare that they have no competing interests.

## Authors' contributions

FRM was the primary clinician and author. JPH helped in the preparation of the manuscript and discussions regarding the topic. FRM and JPH read and approved the final manuscript.
